# Functional imaging guided stereotactic ablative body radiotherapy (SABR) with focal dose escalation and bladder trigone sparing for intermediate and high-risk prostate cancer: study protocol for phase II safo trial

**DOI:** 10.1186/s13014-024-02440-7

**Published:** 2024-05-03

**Authors:** Almudena Zapatero, Pablo Castro, María Roch, Pablo Rodríguez Carnero, Sara Carroceda, Alexandra Elena Stoica Rosciupchin, Sergio Honorato Hernández, Leopoldo Cogorno, Alfonso Gómez Iturriaga, David Büchser García

**Affiliations:** 1 Radiation Oncology Department, Hospital Universitario de la Princesa, Health Research Institute IIS- IP, Diego de León 62, 28006 Madrid, Spain; 2 Medical Physics Department, Hospital Universitario de la Princesa, Health Research Institute IIS- IP, Madrid, Spain; 3 Radiology Department, Hospital Universitario de la Princesa, Health Research Institute IIS- IP, Madrid, Spain; 4 Urology Department, Hospital Universitario de la Princesa, Health Research Institute IIS- IP, Madrid, Spain; 5 Department of Surgery and Radiology and Physical Medicine, Hospital Universitario Cruces, University of the Basque Country UPV/EHU, Biobizkaia Health Research Institute, Bizkaia, Spain

**Keywords:** Prostate cancer, High-risk prostate cancer, SABR, SBRT, Ultrahypofractionation, Extreme hypofractionation, Magnetic resonance imaging, MRI, Focal boost, Biochemical control

## Abstract

**Background:**

Stereotactic ablative body radiotherapy (SABR) is an emerging treatment alternative for patients with localized low and intermediate risk prostate cancer patients. As already explored by some authors in the context of conventional moderate hypofractionated radiotherapy, focal boost of the index lesion defined by magnetic resonance imaging (MRI) is associated with an improved biochemical outcome. The objective of this phase II trial is to determine the effectiveness (in terms of biochemical, morphological and functional control), the safety and impact on quality of life, of prostate SABR with MRI guided focal dose intensification in males with intermediate and high-risk localized prostate cancer.

**Methods:**

Patients with intermediate and high-risk prostate cancer according to NCCN definition will be treated with SABR 36.25 Gy in 5 fractions to the whole prostate gland with MRI guided simultaneous integrated focal boost (SIB) to the index lesion (IL) up to 50 Gy in 5 fractions, using a protocol of bladder trigone and urethra sparing. Intra-fractional motion will be monitored with daily cone beam computed tomography (CBCT) and intra-fractional tracking with intraprostatic gold fiducials. Androgen deprivation therapy (ADT) will be allowed. The primary endpoint will be efficacy in terms of biochemical and local control assessed by Phoenix criteria and post-treatment MRI respectively. The secondary endpoints will encompass acute and late toxicity, quality of life (QoL) and progression-free survival. Finally, the subgroup of high-risk patients will be involved in a prospective study focused on immuno-phenotyping.

**Discussion:**

To the best of our knowledge, this is the first trial to evaluate the impact of post-treatment MRI on local control among patients with intermediate and high-risk prostate cancer undergoing SABR and MRI guided focal intensification. The results of this trial will enhance our understanding of treatment focal intensification through the employment of the SABR technique within this specific patient subgroup, particularly among those with high-risk disease, and will help to clarify the significance of MRI in monitoring local responses. Hopefully will also help to design more personalized biomarker-based phase III trials in this specific context. Additionally, this trial is expected to be incorporated into a prospective radiomics study focused on localized prostate cancer treated with radiotherapy.

**Trial registration:**

Clinicaltrials.gov identifier: NCT05919524; Registered 17 July 2023.

**Trial Sponsor:**

IRAD/SEOR (Instituto de Investigación de Oncología Radioterápica / Sociedad Española de Oncología Radioterápica).

**Study setting:**

Clinicaltrials.gov identifier: NCT05919524; Registered 17 July 2023.

**Trial status:**

Protocol version number and date: v. 5/ 17 May-2023. Date of recruitment start: August 8, 2023. Date of recruitment completion: July 1, 2024.

## Background

Dose intensification has shown to potentially improve long-term control, particularly in intermediate and high-risk prostate cancer patients. Hence, it is crucial to intensify efforts in minimizing the dose to organs at risk. Employing advanced image-guided radiation therapy (IGRT) techniques and optimizing dosage to the rectum and urinary structures has become essential to accomplish this objective.

Focal boost to index lesions (ILs) represents an interesting strategy for dose escalation. This approach is based on the clinical and pathologic evidence showing that the ILs might be the nucleus of the tumor aggressiveness and post-treatment recurrence [1, 2].The clinical benefits of and tolerance of focal boost to the ILs have been previously reported [3]. The oncological benefit was demonstrated in the phase 3 FLAME trial [4] where patients were randomized to receive standard radiation therapy of 77 Gy in 35 fractions, with or without a focal boost to 95 Gy. Results indicated an improved biochemical-free survival with the IL boost at 5-years, with no adverse impact on toxicities and quality of life (QoL). Other studies have also reported excellent morphological and functional results with highly selected focal radiation dose intensification [5].

Nevertheless, it remains uncertain whether the benefit of ILs boost persist in the context of ultrahypofractionated radiation therapy (UHRT). In this setting, several phase I/II studies have presented preliminary findings on early toxicities with a focal boost ranging from 40 to 55 Gy over 5-fraction prostate stereotactic ablative radiotherapy (SABR) [6–8].

However, most of the evidence with UHRT has been reported in low- and intermediate-risk prostate cancer, while there is very limited data in high-risk disease [9]. At present, the largest available randomized evidence for UHRT comes from HYPO-RT-PC [10], a non-inferiority phase III clinical trial that randomized 1,200 prostate cancer patients to UHRT (42.7 Gy in 7 fractions) versus conventional fractionated radiation therapy (78 Gy in 39 fractions)—including 126 high-risk patients. Although equally effective, UHRT was however related with increased urinary toxicity that could potentially been lower if a genuine SABR technique had been employed. Other reported prospective studies of SABR in high-risk prostate cancer are small size phase II trials with preliminary results [9, 11]. An individual patient meta-analysis of 344 patients with high-risk prostate cancer treated with SABR has shown promising efficacy [12]. Further controlled prospective studies are needed to verify these results and investigate the optimal dose and target volume in this scenario.

The present study is a prospective single arm phase II (proof of concept) trial designed to evaluate the effectiveness, safety and impact on quality of life of focal dose intensification to ILs using SABR technology and UHRT in intermediate and high-risk prostate cancer patients. The hypothesis of this study is that the combination of prostate SABR with imaging guided focal dose intensification on the ILs, together with partial preservation of the prostatic urethra and bladder trigone, would lead to a higher probability of local control without a significant increase in toxicity or even lower complications compared to standard clinical practice. The results will help determine the design of subsequent based phase III trials in the exclusive SABR setting in high-risk disease, the value of MRI in monitoring response, and hopefully the impact of immune phenotyping in the individualized approach of SABR dose intensification in localized prostate cancer.

## Methods: design, participants, interventions and outcomes/design

This is a prospective single arm phase II (proof of concept) trial, approved by the Ethics Committee of the Hospital Universitario de La Princesa, Health Research Institute in Madrid (2023.5222) and registered on clinicaltrials.gov (NCT05919524). To ensure and adequate enrolment in accordance with the chronogram, all patients with localized prostate cancer will initially be visited by the trial investigators.

The aim is to evaluate the effectiveness, safety and impact on quality of life of MRI guided focal dose intensification to ILs using SABR technology and UHRT in intermediate and high-risk prostate cancer patients including partial sparing of the bladder trigone and urethra. An interim analysis in the first 5 cases will be carried out by the PI to assess the safety of the trial, through the analysis of acute urinary and rectal toxicity as a surrogate variable for late toxicity. This analysis will be accessible to all investigator and the sponsor of the trial.

The high-risk patients in this study will be prospectively included (following screening and informed consent) in a translational study of biomarkers (Immune Phenotype Of Metastatic Prostate Cancer: Immuno-PROfiling; grant from Health Research Fund -FIS- PI21/01111).

### Objectives

#### Co-primary endpoints and measures

- Biochemical progression-free survival, from time of inclusion until biochemical failure defined by the Phoenix Consensus Conference recommendation from the Radiation Therapy Oncology Group - American Society for Radiation Oncology (RTOG-ASTRO): A rise by 2 ng/ml or more above the nadir prostate specific antigen (PSA) confirmed by a second observation taken 3–4 weeks later.
- Local control defined as the disappearance of suspicious images (ILs) on mpMRI performed 6–9 months after the end of radiotherapy.

#### Secondary endpoints and measures

- Incidence and severity of acute urinary treatment-related adverse events graded according to CTCAE (Common Terminology Criteria for Adverse Events) v5.0 scale. Every urinary event occurring within 3 months from treatment completion will be defined as “acute event”. All adverse events will be recorded and graded according to CTCAE V5.0 scale (graded from 0 to 5 with greater values representing worse outcomes) [Time Frame: 90 days].
- Incidence and severity of acute rectal treatment-related adverse events graded according to CTCAE (Common Terminology Criteria for Adverse Events) v5.0 scale.
- This paragraph follows the prior. Every rectal event occurring within 3 months from treatment completion will be defined as “acute event”. All adverse events will be recorded and graded according to CTCAE V5.0 scale (graded from 0 to 5 with greater values representing worse outcomes) [Time Frame: 90 days].
- Incidence and severity of late urinary treatment-related adverse events graded according to CTCAE (Common Terminology Criteria for Adverse Events) v5.0 scale. Every urinary event occurring after 3 months from treatment completion will be defined as “late event”. All adverse events will be recorded and graded according to CTCAE V5.0 scale (graded from 0 to 5 with greater values representing worse outcomes) [Time Frame: 2 years].
- Incidence and severity of late rectal treatment-related adverse events graded according to CTCAE (Common Terminology Criteria for Adverse Events) v5.0 scale. Every rectal event occurring after 3 months from treatment completion will be defined as “late event”. All adverse events will be recorded and graded according to CTCAE V5.0 scale (graded from 0 to 5 with greater values representing worse outcomes) [Time Frame: 2 years].
- Patient reported outcomes and quality of life assessment. Impact on Quality of life affecting the genitourinary, gastrointestinal, sexual and hormonal domains using the EPIC-26 short form, the International Prostate Symptom Score (IPSS) and the Expanded Prostate Index Composite-26. [Time Frame: 2 years]

### Eligibility criteria

#### Inclusion criteria

- Signed written informed consent obtained by the investigators of this study according to ICH/GCP regulations before registration and prior to any trial procedure.
- ECOG 0–1
- Minimum age 18 years.
- Histologically confirmed adenocarcinoma of the prostate.
- Primary localized Prostate cancer, cN0 and cM0, intermediate or high-risk disease according to NCCN 2023.
- Tumor clinical stage cT2-T3a with visible index lesion on pretreatment MRI. MRI pre-treatment is mandatory.
- Desirable prostate volume in MRI (not mandatory) < 80 cc or > 80 cc if urinary function is preserved and is dosimetrically feasible.
- IPSS (International Prostate Symptom Score) ≤ 18; alfa blockers allowed.

#### Exclusion criteria

- Unresolved previous prostatitis, symptomatic urethral stenosis.
- Bilateral hip prosthesis.
- Previous surgery at the prostate level (transurethral resection of the prostate or adenomectomy) within the past 6 months.
- Prior pelvic RT.
- Evidence of T3b or T4 clinical stage or N1 or M1 (presence of distant metastases).
- Severe or active co-morbidity likely to impact on the advisability of SABR.
- Previous malign neoplasia unless in remission for at least 3 years from registration with the exception of non-melanoma skin cancer.

### Intervention

#### Radiation dose

- 36.25 Gy in 5 sessions of 7.25 Gy (EQD2 of 85 Gy with an alpha/beta of 1.5) to the whole prostate gland + seminal vesicles + margin (planned target volume1, PTV1), every other day. Weekly sessions will be permitted on an exceptional basis for selected patients with a prostate volume exceeding 80 cc and IPSS values ranging between 15 and 18 (without adjusting for overall treatment duration).
- Simultaneous “isotoxic” focal boost of up to 50 Gy in 5 sessions to the index lesion + margin (PTV2), prioritizing critical organ restriction criteria at all times.
- Additional urethra and bladder trigone sparing constrains.

### Protocol design and procedures

Once adenocarcinoma of the prostate is histologically confirmed, patients will be staged with multiparametric MRI of the prostate consisting of T2 in axial, coronal and sagittal planes, axial T1 non-contrast of the pelvis, diffusion weighted imaging (DWI), and T1 dynamic contrast-enhanced sequences (DCE), using a body-phased array coil. The acquisition and imaging protocol will be consistent with the European Society of Urogenital Radiology recommendations [13]. A prostate imaging reporting and data system (PI-RADS version 2.1) score of at least 3 is required for the lesion to be deemed appropriate for focal intensification, with confirmatory target biopsies conducted on equivocal MRI findings. The MRI images acquired will serve as the substrate for patient inclusion in the study and will provide a reference basis for the subsequent definition of treatment volumes (prostate gland and ILs) in treatment planning.

After obtaining informed consent, a minimum of three gold fiducial seeds will be implanted in the prostate through ultrasound-guided trans-rectal pre-loaded needles, along with recto-prostatic spacer at physician discretion (optional). Between 7 and 15 days after fiducial implantation, a computed tomography (CT, slice thickness of 2 mm) and a new MRI study of the prostate that included axial T2 weighted images, DWI and a T2* gradient recalled echo images will be performed in radiotherapy supine position (flat table top with an indexed knee and ankle immobilization device) for treatment planning following a comfortably full bladder and an empty rectum protocol. To help with the contouring of the urethra, a 12 French Foley non-radiopaque catheter will be inserted before the CT simulation. A matching point registration (based on fiducials) of planning CT and MRI will be used for prostate and tumor delineation. In those patients treated with neoadjuvant hormonotherapy for more than 3 months, we will use a second diagnostic mpMRI registration to add prostate and tumor delineation.

The clinical target volume (CTV) will include the prostate and one-third of seminal vesicles for favorable intermediate risk and two-third of seminal vesicles for unfavorable intermediate and high-risk patients. A margin of 3 mm posteriorly and 5 mm in all the other directions will be added to create the planning target volume for the prostate (PTV-P) and 2–3 mm in all directions for the mpMRI visible index lesion (PTV-IL), except for interfase GTV-rectum/urethra that will be (0–1 mm). ESTRO ACROP consensus guideline for CT-MRI target volume delineation will be used for contouring [14]. Organs at risk (OAR) will be contoured according to RTOG guidelines and will include the bladder and rectum as solid organs, the rectum PRV (rectum + margin 2–3 mm), the urethra PRV (Foley catheter with a 1–2 mm isotropic ring expansion), the bulbar urethra, the bladder trigone [15] and femoral heads. Table 1 summarizes the dose constraints for the OAR. Indeed, the criteria for further excluding the patient from the trial is not the location of the IL, but rather the consideration of mandatory organ-at-risk (OAR) constraints.

**Table 1:** Summary of time schedules, events and follow-up

Study Procedure	Screening visit	During SABR Protocol treatment		Follow-up		
		During SABR treatment	End of SABR treatment	Year 1–2 First month and every 3months	Years 3 Every 6 months	End of study
Medical history and physical examination	X			X	X	X
Informed consent	X					
Concomitant medications	X		X	X	X	X
Bood counts, Serum chemistry PSA/Testosterona	X			X	X	X
Urinary or rectal comorbidities	X	X	X	X	X	X
Adverse events	X	X	X	X	X	X
Prostate MRI	X			X	X*	
Standard imaging work up	X#					
PET–TC (PSMA or Choline in High Risk	X#					
QoL questionaires	X		X	X	X	X
Urinary and rectal events and grades	X	X	X	X	X	X
Survival	X		X	X	X	X

*: at 3 months and 6–12 months when required

#: At screening and when required

### Dose prescription, treatment delivery and quality control

Radiation treatment will consist on 5 fractions of 7.25 Gy (every other day) to the whole prostate gland and the seminal vesicles with a simultaneous “isotoxic” focal boost of up to 50 Gy in 5 sessions to the ILs, prioritizing critical organ restriction criteria at all times. We have selected this dose schedule to further escalate dose to the tumor lesion to an EQD2Gy (equivalent dose in 2 Gy fractions) above 125–150 Gy (alfa/β 1.5), while maintaining an EQD2Gy of 85 Gy to the whole prostate.

The treatment will be administered using an electron Linear Accelerator (Linac) named TrueBeam with Volumetric Modulated Arc Therapy (VMAT) technique employing complete arcs of 36 degrees. The treatment design will be conducted using an inverse optimization process integrated into specific PC-software (Treatment Planning System, TPS) that models the mechanical and dosimetric properties of the linac. The procedure will be conducted with filter-free energy to minimize treatment times as much as possible, utilizing the highest available energy, specifically 10 FFF (Flattening Filter Free), and a maximum rate of 2400 MU/min (Monitor Units per minute). The fiducial markers will serve as a reference for daily mobility control IGRT prior treatment using cone beam CT (CBCT) technology, and intra-fractional tracking during treatment using KV acquisitions every 20º of arc rotation. Images acquired daily through pre-treatment and during-treatment will help monitor and quantify uncertainties associated with positioning, allowing assessment of treatment volume safety margins. Also on a daily basis, a verification of the isocenter position of the kV On-Board Imager (OBI) imaging system will be performed using the Machine Performance Check device with a tolerance of 0.5 millimeters.

An alternative calculation to the dose distribution provided by the TPS will be performed prior to treatment. The discrepancy in point dose should not exceed 3%. Additionally, in the comparison between dose distributions, it will be ensured that 95% of the analyzed points meet the gamma criterion of 3%/3 mm less than 1. Furthermore, it will be verified that the linac delivers the treatment correctly and that dose distributions are equivalent to those calculated by the TPS. This verification is carried out using a high-resolution detector array.

### Systemic therapy

Short- (6 months) or long-term (18–28 months) androgen derivation therapy (ADT) will be allowed for unfavourable intermediate and high-risk tumours, respectively, in agreement with clinical guidelines and following a protocol of risk adapted ADT. Since the publication in 2022 of the results of the meta-analysis from the STAMPEDE platform [16], we also incorporate doublet with ADT and Abiraterone-prednisone in selected high and very high-risk patients.

### Outcomes: clinical and toxicity assessment and follow-up

Clinical assessment is planned at baseline, weekly during radiation treatment, at 3, 6, 9 and 12 months follow-up and every 6 months thereafter until 5 years. Prostate-specific antigen (PSA) values, serum testosterone level and a complete blood count will be obtained at each visit. Biochemical control will be assessed through PSA measurements. A post-radiotherapy MRI study is mandatory for local control assessment, and is planned at six months following radiation treatment and repeated 3–6 months later in those cases in which a complete response is not achieved. The high-risk patients in this study will be prospectively included (following screening and informed consent) in a translational study of biomarkers (Immune Phenotype Of Metastatic Prostate Cancer: Immuno-PROfiling; grant from Spanish Health Research Fund -FIS- PI21/01111). Toxicity and perceived quality of life in the urinary, digestive, and hormonal domains will be evaluated using specific assessment scales (EORTC/RTOG and CTCAEs) and validated specific questionnaires (IPSS/EPIC 26) prior to treatment and during follow-up (Table 2). Prostate biopsy will be performed in clinically selected cases according to standard practice.

**Table 2:** Summary of dose constrains

Structure	Dosimetric parameter	Constrains per protocol
PTV1	D98% (Dnear min)	> 95% (34.44 Gy)
CTV1– PRV Urethra	D98% (Dnear min)	> 95% (34.44 Gy)
	D95% (Dnear min)	> 100% (36.25 Gy)
PTV2	V95% (47.5 Gy)	> 95%
	V105% (52.5 Gy)	< 5%
Rectum	Maximum dose (0.03 cc)	≤ 39 Gy
	Median dose	≤ 18.1 Gy
	V18Gy	≤ 50%
	V29Gy	≤ 20%
	V36Gy	< 1 cc (alternative 2 cc)
PRV Rectum	Maximum dose (0.03 cc)	≤ 41 Gy
Bladder	Maximum dose (0.03 cc)	≤ 39 Gy
	V18.1 Gy	≤ 40%
	V36Gy	≤ 10%
	V37Gy	< 5 cc (alternative 10 cc)
PRV Urethra	Maximum dose (0.03 cc)	≤ 35.25 Gy (alternative 36.25 Gy)
Bulbar Urethra	Maximum dose (1 cc)	≤ 40 Gy
Bladder trigone	Maximum dose (0.1 cc)	≤ 38 Gy
Sigma Bowel	V20Gy	< 1%
Penile bulb	Maximum dose (0.03 cc)	< 40 Gy
	V21.6 Gy	< 3 cc
Femoral Heads	V14.5 Gy	≤ 5%
	Maximum dose (0.03 cc)	< 32.4 Gy

### Statistical analysis

#### Sample size

Based on limited published results of SABR in high-risk patients, the aim of this trial is to demonstrate that the success rate (increase biochemical free survival) using focal intensification in high-risk prostate cancer, will reach 85%, with a minimum of 65%. Since the goal is to establish that the success rate exceeds a pre-established lower limit, this is a non-inferiority study, employing a one-sided analysis. The assumed success rate in the sample is 0.85. Using alpha and beta levels of 0.05 and 0.2 (contrast power of 0.8) and a one-sided test, a sample of 23 patients will be required to demonstrate that the lower limit of the 95% confidence interval for the success rate is greater than or equal to 0.50. The G*Power 3.1 software was employed for the calculations. It is expected that the recruitment target of 27 patients, accounting for a 15% margin for potential losses, will be easily met within the estimated two-year recruitment period. Depending on preliminary results, a decision and amend will be consider to extend the enrollment to achieve recruitment goal of 50 patients and a 5 years endpoint analysis.

### Definitions and statistical analysis

The co-primary endpoints are the biochemical-disease-free survival and the MRI- defined local control. Biochemical failure was defined according to the Phoenix definition (PSA > 2 ng/mL above the currently observed PSA nadir). An image complete response is defined as disappearance of all morphological and functional lesions in MRI 6 to 12 months after radiotherapy.

No confirmatory biopsy is required. Secondary endpoints included acute and late toxicity and QoL assessment. Urinary and rectal toxicity will be calculated using the maximal recorded toxicity per patient and date of occurrence for actuarial calculation.

Descriptive statistics will be used to summarize demographic and tumor and treatment characteristics as well as acute and late toxicity and QoL outcomes. The Kaplan-Meier method will be used to determine biochemical relapse free and toxicity-free survival rates and medians and the log-rank test for survival rates. The chi2 or Fisher exact test will be used to evaluate differences in patient and treatment characteristics for categorical variables. The t-test, analysis of variance, or Mann-Whitney test will be used, depending on the type and distribution of the variables, to evaluate differences in patient and treatment characteristics for continuous data. The three domains of EPIC scores and sub scores (urinary [function/bother], bowel [function/bother] and sexual [function/bother]) and IPSS will be analyzed to estimate differences in QoL score change from baseline, adjusting by baseline score. A decreased of > 0.5 standard deviation (SD) of baseline values for each domain score will be considered clinically relevant (mild change). A change > 1 (SD) of baseline values will be considered moderately relevant (moderate change) and a change of > 2 SD will be considered a severe change. All statistical analysis will be performed with SAS 9.4. P-values less than 5% will be considered as significant.

## Discussion

There is substantial clinical evidence backing the escalation of radiation therapy doses, demonstrating enhanced biochemical disease-free survival in patients with prostate cancer across all risk groups. This improvement is particularly notable in those with intermediate and high-risk tumors [17, 18]. The utilization of UHRT employing the SABR technique, which enables the delivery of elevated radiation doses in 5 or fewer fractions, has proven to be a secure, efficient, and convenient approach in treating clinically localized prostate cancer. SABR has been predominantly explored in low- and intermediate-risk prostate cancer patients, yielding excellent outcomes [10, 19, 20, 21] However, several interrogations persist concerning the optimal dose, target volumes and timing. Interestingly, a relevant question that needs further exploration is the effectiveness of SABR in high-risk patients, especially given the limited number of studies addressing this particular aspect [7, 9, 11, 12, 22–24].

Given the potential for dose escalation to improve long-term disease control even in the context of extreme hypofractionation, efforts to further minimize doses to organs at risk and enhance overall tolerance should be actively pursued. Based on pathological investigations that suggest that the presence of IPLs serves as a robust indicator of tumor aggressiveness and post-radiotherapy local recurrence [1, 2], a focal dose boost to IPLs has been proposed as a potential way of individualized dose intensification aimed to improve local control and increase biochemical disease-free survival (bDFS) without compromising the sparing of organs at risk (OARs). Several contemporary studies [3, 4], have demonstrated the feasibility and applicability of this treatment, along with good safety and patient tolerance. We recently reported an excellent morphological and functional MRI response in a phase II trial of MRI-guided focal boost using VMAT hypofractionated technique [5]. The oncological benefit of focal DIL boost is now confirmed in the phase 3 FLAME trial that has shown improved biochemical-free survival with IL boost at 5 years, without affecting toxicities and QoL [4].

Since the value of dose intensification in high-risk prostate cancer is well established [17, 18, 25], it becomes evident that the next step should include the exploration of the advantages of both, UHRT with SABR and an individualized focal dose intensification on index lesions.

In this phase II proof of concept trial, we aimed to assess whether the integration of three strategies -UHRT SABR with focal dose intensification on the prostatic IL and a preservation of the prostatic urethra and bladder trigone-, would lead to a higher probability of local control in intermediate and high-risk prostate cancer patients, without a significant increase in toxicity compared to standard clinical practice. To our knowledge this is the first trial to assess the impact on local control and monitoring of post treatment MRI. This trial is also anticipated to be included in a prospective radiomics study focused in prostate cancer treated with radiotherapy. The information derived from this trial together with the results of the sub analysis of biomarkers in high risk disease, will help to design more personalized designs of subsequent phase III trials in the exclusive RT setting for the treatment of high-risk disease.

**Trial Sponsor** IRAD/SEOR (Instituto de Investigación de Oncología Radioterápica / Sociedad Española de Oncología Radioterápica).

## Data Availability

The datasets used and/or analysed during the current study are available from the corresponding author on reasonable request.

## Abbreviations

ADT: Androgen deprivation therapy
bDFS: biochemical disease-free survival
CBCT: cone beam computed tomography
CT: Computerized tomography
CTCAE: Common Terminology Criteria for Adverse Events
CTV: Clinical target volume
EPIC 26: Expanded Prostate Index Composite-26
EQD2: Equivalent total doses in 2-Gy fractions
ESTRO/ACROP: European Society for Radiotherapy and Oncology/ Advisory Committee for Radiation Oncology Practice
IGRT: Image-Guided Radiation Therapy
IL: Index lesion
IPSS: International Prostate Symptom Score
MRI: magnetic resonance imaging
NCCN: National Comprehensive Cancer Network
OAR: Organs at risk
PRV: Planning organ at risk volume
PSA: Prostatic specific antigen
PTV: Planning target volume
QoL: quality of life
RTOG-ASTRO: Radiation Therapy Oncology Group - American Society for Radiation Oncology
SABR: Stereotactic ablative radiation therapy
SBRT: Stereotactic body radiation therapy
SD: standard deviation
SIB: Simultaneous integrated focal boost
UHRT: Ultra hypofractionated radiation therapy
VMAT: Volumetric Modulated Arc Therapy
